# Papillary Thyroid Carcinoma with Desmoid-Like Fibromatosis: Double Trouble?

**DOI:** 10.1007/s12022-022-09735-z

**Published:** 2022-10-15

**Authors:** C. Christofer Juhlin, Martin Hysek, Adam Stenman, Jan Zedenius

**Affiliations:** 1 Department of Pathology and Cancer Diagnostics, Karolinska University Hospital, 176 64, P1:02 Solna, Stockholm, Sweden; 2 Department of Breast, Endocrine Tumors and Sarcoma, Karolinska University Hospital, Stockholm, Sweden

**Keywords:** Papillary thyroid carcinoma, Desmoid-type fibromatosis, BRAF, Beta-catenin

## Case History

The patient was a 39-year-old female with a family history of non-specified pancreatic cancer but without previous medical history or hereditary thyroid disorders. She presented with a palpable nodule in her right thyroid lobe, and fine-needle aspiration cytology was consistent with a Bethesda VI category. A total thyroidectomy with a central lymph node dissection was performed.

## What Is Your Diagnosis?

**Fig. 1:**
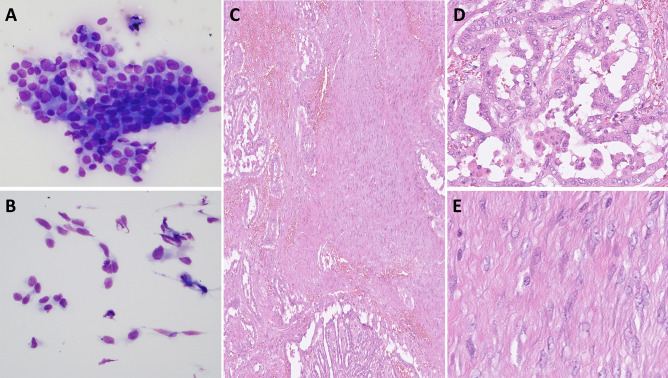
Fine-needle aspiration biopsy (May-Grunwald-Giemsa stain) consistent with a Bethesda VI category, represented by solid to papillary formations of epithelial tumor cells with papillary thyroid carcinoma (PTC)-related nuclear atypia (**A**). Areas with aggregated spindle cells were also noted (**B**). Histology (hematoxylin and eosin stain) displayed a biphasic tumor with an epithelial component arranged in papillary structures intermixed with a dense network of spindle cells (**C**). The epithelial component displayed florid PTC-related nuclear changes (**D**), while the spindle cells were bland-looking (**E**)

**Fig. 2:**
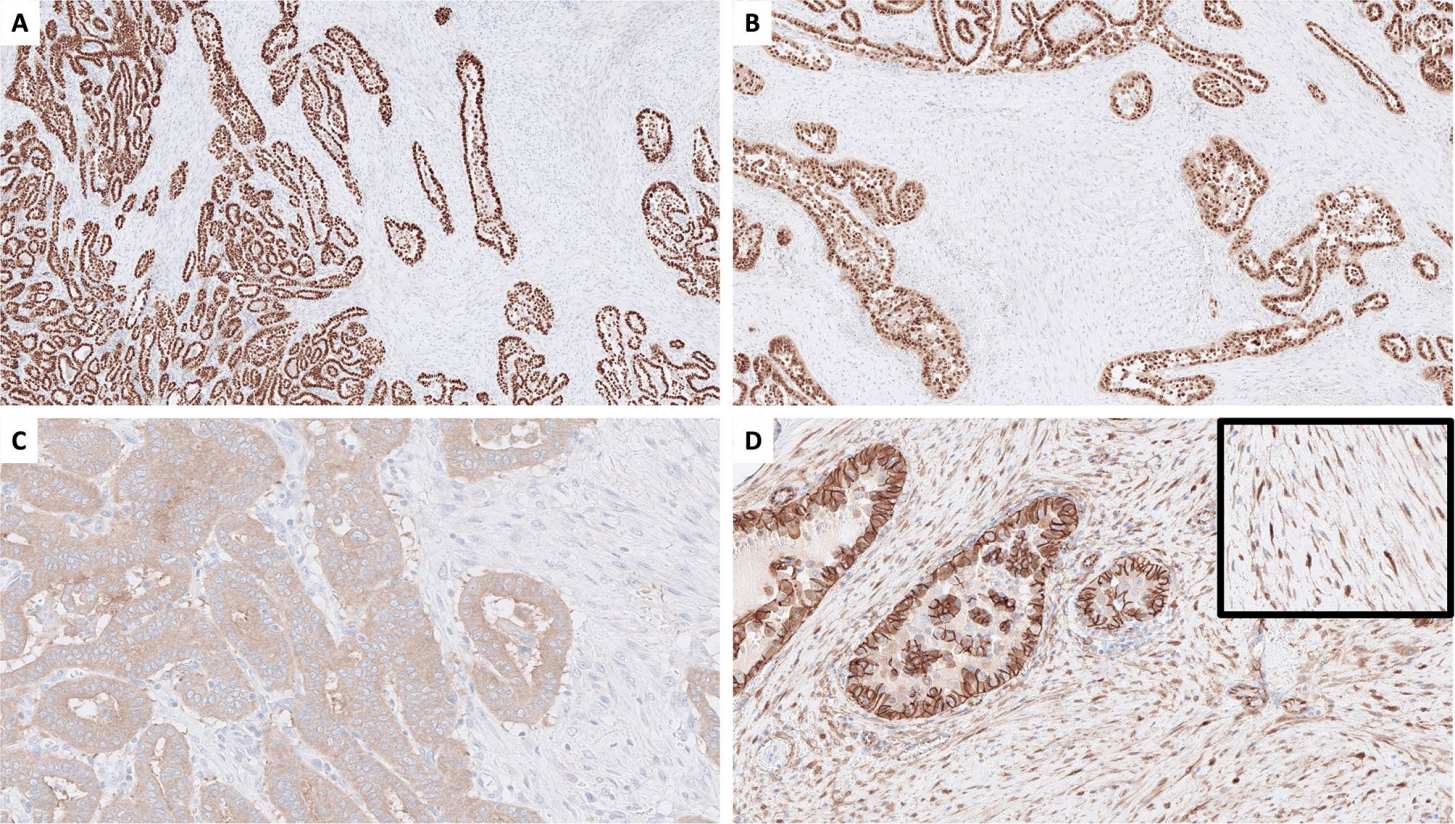
Immunohistochemistry displayed TTF1 (**A**), monoclonal PAX8 (**B**), and VE1 (**C**) positivity in the papillary thyroid carcinoma (PTC) component, while the desmoid-type fibromatosis (DTF) component was negative for all markers except nuclear beta-catenin expression (**D**), with a high magnification insert

Figure composites (see Figs. 1 and 2).

## Diagnosis: Papillary Thyroid Carcinoma with Desmoid-Type Fibromatosis

Preoperatively, the fine-needle aspiration cytology was consistent with a papillary thyroid carcinoma (PTC) (Bethesda VI category), thus favoring malignancy (Fig. 1A). Odd aggregations of spindle cells were also identified (Fig. 1B). Grossly, a 41 mm thyroid mass with white to grey colored cut surface was seen in the right lobe. Histologically, there was a biphasic neoplasm consisting of papillary and focal follicular structures with cells displaying florid PTC nuclear atypia intermingled with a cellular stroma built-up by spindle cells without high-grade features (necrosis and/or ≥ 5 mitoses per 2 mm^2^) (Fig. 1C–E). There was neither extrathyroidal extension nor angioinvasion or perineural invasion. By immunohistochemistry, the epithelial component showed positivity for TTF1 (SP141 clone), monoclonal PAX8 (MRQ-50 clone), thyroglobulin, and *BRAF* p.V600E mutation-specific VE1 antibody (Fig. 2A–C). The mesenchymal component was positive for smooth muscle actin (not shown) and nuclear beta-catenin (Fig. 2D). A single 1.1 mm nodal metastasis of the epithelial component was noted. The patient was discussed at a multidisciplinary tumor board meeting, and the tumor was staged as pT3aN1a (high risk according to ETA guidelines and intermediate risk according to ATA). She received a postoperative radioiodine (RAI) dose of 3.7 gigabecquerel and is currently recurrence-free with a limited follow-up time of 5 months.

## Comment

A plethora of histological PTC subtypes exists, many of them with distinct correlations to underlying genetics and prognosis [1]. PTC with desmoid-type fibromatosis (PTC-DTF) is an exceedingly rare subtype consisting of two distinct components, a mainly *BRAF* p.V600E-mutated PTC intermingled with a *CTNNB1* driven soft tissue neoplasm [2, 3]. To date, only around 50 cases have been reported in the literature [4]. The tumor has previously been described as “PTC with nodular fasciitis-like stroma,” but this nomenclature is no longer endorsed in the new WHO classification given the distinct characteristics of this disease [3]. The exact proportions of the PTC and DTF components are not established for this entity, and the extent of the desmoid-type stroma may vary in individual cases [5]. Interestingly, most PTC-DTF cases are categorized as Bethesda V-VI lesions and are rarely described as schwannoma or fibroma [5]. The histological diagnosis of PTC-DTF requires the use of appropriate immunohistochemical biomarkers, as illustrated in this case. The VE1 antibody helps highlight the PTC component, and the nuclear accumulation of beta-catenin in the DTF component also helps to distinguish this entity from PTCs with a reactive, desmoplastic stroma [3].

As PTC-DTF is infrequently encountered, little is known regarding the association to clinical parameters and patient outcomes. While distant metastases have not been defined yet, PTC-DTFs often display locally infiltrative disease (extrathyroidal extension into strap muscle or adjacent structures) as well as central and lateral lymph node metastases [5]. Interestingly, while most nodal metastases only contain the PTC component, subsets of cases display nodal metastases including both the PTC and DTF components [5]. The PTC component seems to be RAI avid, and the mesenchymal component is believed to lack RAI avidity. Other adjuvant oncologic therapies have also been used, but the rarity of PTC-DTF makes any correlations to patient outcome unreliable [4]. However, pathologists should be aware of this rare subtype of PTC given the unique characteristics of these neoplasms to enable their distinction from other entities.

## Data Availability

The authors confirm that the data supporting the findings of this study are available within the article.
